# Effects of Aging on Endotoxin Tolerance Induced by Lipopolysaccharides Derived from *Porphyromonas gingivalis* and *Escherichia coli*

**DOI:** 10.1371/journal.pone.0039224

**Published:** 2012-06-18

**Authors:** Ying Sun, Hui Li, Mi-Fang Yang, Wei Shu, Meng-Jun Sun, Yan Xu

**Affiliations:** 1 Institute of Stomatology, Nanjing Medical University, Nanjing, China; 2 Department of Periodontology, Stomatology Hospital affiliated to Nanjing Medical University, Nanjing, China; Charité, Campus Benjamin Franklin, Germany

## Abstract

**Background:**

Periodontitis is a bacterially induced chronic inflammatory disease. Exposure of the host to periodontal pathogens and their virulence factors induces a state of hyporesponsiveness to subsequent stimulations, termed endotoxin tolerance. Aging has a profound effect on immune response to bacteria challenge. The aim of this study was to explore the effects of aging on endotoxin tolerance induced by *Porphyromonas gingivalis* (*P. gingivalis*) lipopolysaccharide (LPS) and *Escherichia coli* (*E. coli*) LPS in murine peritoneal macrophages.

**Methodology/Principal Findings:**

We studied the cytokine production (TNF-αand IL-10) and Toll-like receptor 2, 4 (TLR2, 4) gene and protein expressions in peritoneal macrophages from young (2-month-old) and middle-aged (12-month-old) ICR mice following single or repeated *P. gingivalis* LPS or *E. coli* LPS stimulation. Pretreatment of peritoneal macrophages with *P. gingivalis* LPS or *E. coli* LPS resulted in a reduction in TNF-α production and an increase in IL-10 production upon secondary stimulation (p<0.05), and the markedly lower levels of TNF-α and higher levels of IL-10 were observed in macrophages from young mice compared with those from middle-aged mice (p<0.05). In addition, LPS restimulations also led to the significantly lower expression levels of TLR2, 4 mRNA and protein in macrophages from young mice (p<0.05).

**Conclusions/Significance:**

Repeated LPS stimulations triggered endotoxin tolerance in peritoneal macrophages and the ability to develop tolerance in young mice was more excellent. The impaired ability to develop endotoxin tolerance resulted from aging might be related to TLR2, 4 and might lead to the incontrollable periodontal inflammation in older adults.

## Introduction

Periodontitis is one of the most common oral diseases in humans, which is characterized by the loss of tooth-supporting structures. It is a bacterially induced chronic destructive inflammatory disease and is difficult to treat [1]. Gram-negative bacteria, including *Porphyromonas gingivalis* (*P. gingivalis*), *Prevotella intermedia* (*P. intermedia*), *Fusobacterium nucleatum* (*F. nucleatum*) and *Aggregatibacter actinomycetemcomitans* (*A. actinomycetemcomitans*), have been considered to be the important periodontopathic bacteria. Among them, *P. gingivalis* can be frequently isolated from periodontal pockets in patients with chronic periodontitis, which is the most common form of periodontiis [2], [3]. The cell-wall components of periodontal pathogens, especially lipopolysaccharide (LPS), can trigger a wide range of host responses, including the production of pro-inflammatory cytokines, anti-inflammatory cytokines, and chemokines. Excessive and prolonged immune responses can lead to the destruction of periodontal tissues and may be very important in the progression of periodontitis [4], [5].

Endotoxin tolerance is a phenomenon whereby previous exposure of cells or organisms to microbial products induces a hyporesponsiveness to subsequent challenge and is characterized by diminished release of proinflammatory cytokines, such as TNF-α and IL-1ß [6]. The hyporesponsiveness to a secondary challenge with a different LPS (heterotolerance) is usually weaker than that with the same LPS (homotolerance) [7]. Endotoxin tolerance represents a selective reprogramming aimed at limiting inflammatory damage resulted from activation of the immune system by bacteria or their virulence factors [8]. Therefore, tolerance induced by persistent periodontopathic bacteria stimulations might be essential to maintain homeostasis in periodontal tissues. Accumulating evidence suggested the possible involvement of Toll-like receptors (TLRs) pathways in endotoxin tolerance [9], [10]. However, the exact mechanism, especially for endotoxin tolerance in periodontitis, still remains obscure.

TLRs are type Ι transmembrane proteins found on the surface of mammalian cells and are implicated in the recognition of conserved bacterial cell-wall components [11]. To date, at least 11 human TLRs and 13 murine TLRs have been described. Among them, TLR2 and 4 function as the principal innate sensors for cell-wall components of gram-negative bacteria in mammals [12]–[14], and might be very important in endotoxin tolerance induced by periodontal pathogens [9], [10].

Aging is associated with poor periodontal health and some studies have disclosed the potential relationship between advanced age and the increased prevalence and severity of periodontitis [15], [16]. In old individuals, alterations of both innate and adaptive immunity lead to increased susceptibility to infections, including periodontal inflammation [17], [18]. Age-related changes in the adaptive immune system are well-documented, such as altered cytokine patterns and a decline in Ag-presenting cell function [19]. Researches have also indicated the decreased functions of macrophages, NK cells and lymphocytes with aging, including chemotaxis, phagocytic and scavenger receptor activity, production of reactive oxygen species, the inflammatory wound healing response, and induction of certain cytokine responses [20]–[22]. Macrophages, which play an important role in the innate host response in periodontitis as well as other chronic infections [23], are known to develop endotoxin tolerance [24], [25]. Little is known about the influence of aging on endotoxin tolerance in macrophages. In addition, it is still not fully understood the relationship between age-related alterations in innate immunity and the prognosis of periodontitis.

It is hypothesized that 1) aging might have an effect on endotoxin tolerance, which might be related with the development of periodontitis in aged individuals; and 2) age-related alteration in TLR2, 4 might be associated with the impact of aging on endotoxin tolerance. To better understand the effects of aging on endotoxin tolerance and their underlying mechanisms, endotoxin tolerance was induced by LPS derived from *P. gingivalis* and *E. coli* in peritoneal macrophages from young and middle-aged mice. Then, we explored the production of pro-inflammatory cytokine TNF-α and anti-inflammatory cytokine IL-10 in these cells by enzyme-linked immunosorbent assays (ELISA), and examined the changes of TLR2, 4 expressions by real-time PCR and flow cytometry. Our results revealed the impaired ability to develop endotoxin tolerance resulted from aging, which might have an influence on the development of periodontitis in old individuals. In addition, this impaired ability might be related to the aged-associated changes in TLR2, 4.

## Results

### Cytokine Production in Peritoneal Macrophages upon a Primary or Secondary Exposure to LPS

**Figure 1:**
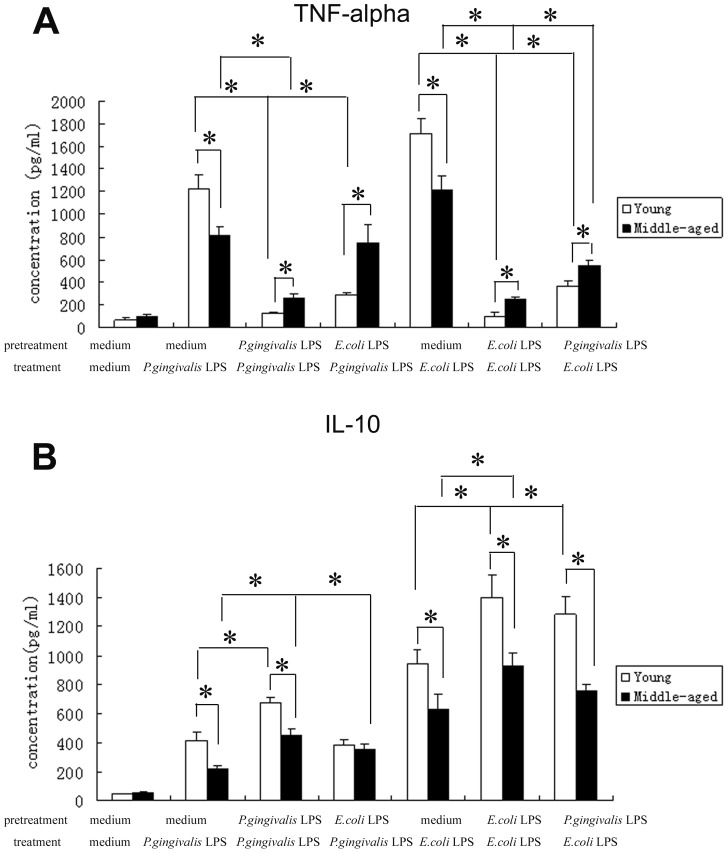
Cytokine production in peritoneal macrophages from young and middle-aged mice stimulated with LPS. Peritoneal macrophages from both young and middle-aged mice were pretreated with medium, 1 µg/ml *P.gingivalis* LPS or 1 µg/ml *E.coli* LPS for 24 h, washed, and then incubated with medium, 1 µg/ml *P.gingivalis* LPS or 1 µg/ml *E.coli* LPS for another 24 h. The levels of TNF-α (1A) and IL-10 (1B) in the culture supernatants were measured by ELISA. Data are expressed as mean±SD (n = 5 per group). *P<0.05.

To explore the secretions of pro-inflammatory cytokine TNF-α and anti-inflammatory cytokine IL-10 by murine peritoneal macrophages, the levels of these cytokines in the culture supernatants were measured by ELISA. Our results revealed that without stimulation, there were no significant differences in the production of all cytokines between peritoneal macrophages from young and middle-aged mice (p>0.05). Stimulations with *P. gingivalis* LPS or *E. coli* LPS for 24 h resulted in marked increases in the levels of TNF-α and IL-10 (p<0.05), and the production of all cytokines secreted by peritoneal macrophages from young mice were significantly higher than those from middle-aged mice (p<0.05). In addition, the secretions of all cytokines induced by *E. coli* LPS were significantly higher than those induced by *P. gingivalis* LPS (p<0.05) (Figure 1).

Next, to investigate the effects of aging on the ability of peritoneal macrophages to develop endotoxin tolerance, cells were pretreated with *P. gingivali*s LPS or *E. coli* LPS for 24 h, then restimulated after washing for an additional 24 h and assayed for cytokine production. When macrophages from both young and middle-aged mice were restimulated with *P. gingivalis* LPS or *E. coli* LPS, significant reductions were observed in the levels of TNF-α compared with those seen following single stimulation (p<0.05), except TNF-α production in the cells from middle-aged mice pretreated with *E. coli* LPS and treated with *P. gingivalis* LPS (p>0.05). Importantly, upon repeated LPS stimulations, the secretions of TNF-α by macrophages from young mice were significantly lower than those from middle-aged mice (p<0.05), which indicated that the ability to develop tolerance to endotoxin in young mice was more excellent. In addition, our study demonstrated that in macrophages from both young and middle-aged mice, homotolerance was much stronger than heterotolerance at the levels of TNF-α (p<0.05) (Figure 1A).

However, the changes of anti-inflammatory cytokine IL-10 levels was not as same as the those of TNF-α. *P. gingivalis* LPS and *E. coli* LPS homotolerance resulted in a significant increase in IL-10 secretions in peritoneal macrophages from both young and middle-aged mice (p<0.05), and the levels of IL-10 secreted by the cells from young mice were significantly higher than those from middle-aged mice (p<0.05). Moreover, in macrophages from young mice, but not middle-aged mice, precondition with *P. gingivalis* LPS and subsequent stimulation with *E. coli* LPS also led to a markedly increased IL-10 production (p<0.05) (Figure 1B).

### Expression of TLR2, 4 in Peritoneal Macrophages after a Primary or Secondary LPS Exposure

**Figure 2:**
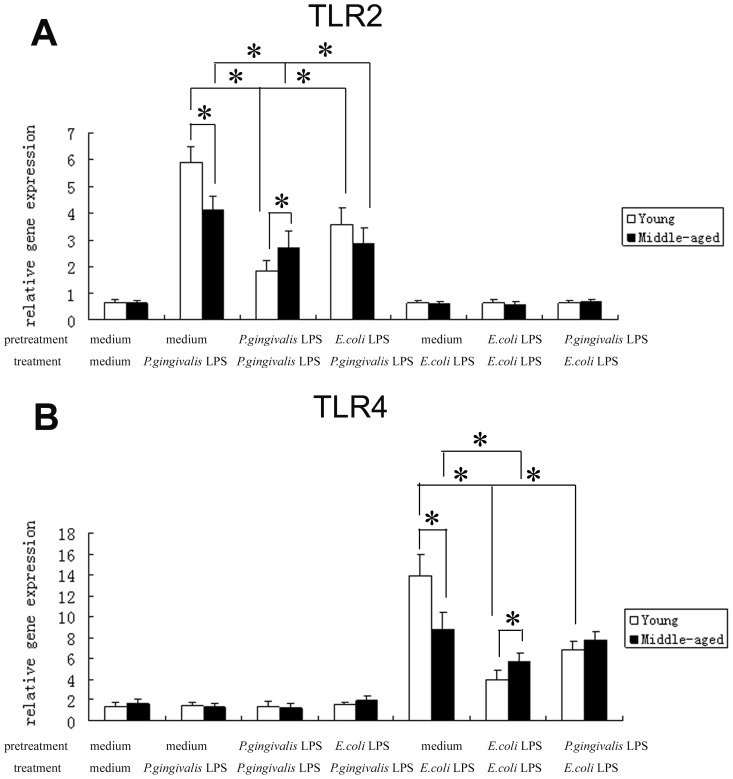
Gene expression changes of TLR2, 4 in peritoneal macrophages from young and middle-aged mice stimulated with LPS. Peritoneal macrophages from both young and middle-aged mice were pretreated with medium, 1 µg/ml *P.gingivalis* LPS or 1 µg/ml *E.coli* LPS for 24 h, washed, and then restimulated with medium, 1 µg/ml *P.gingivalis* LPS or 1 µg/ml *E.coli* LPS for 6 h. Real-time PCR was used to quantify TLR2 (2A) and TLR4 (2B) mRNA expression levels. The absolute mRNA levels of all the genes were normalized to ß-actin levels of individual samples. Data are expressed as mean±SD (n = 5 per group). *P<0.05.

**Figure 3:**
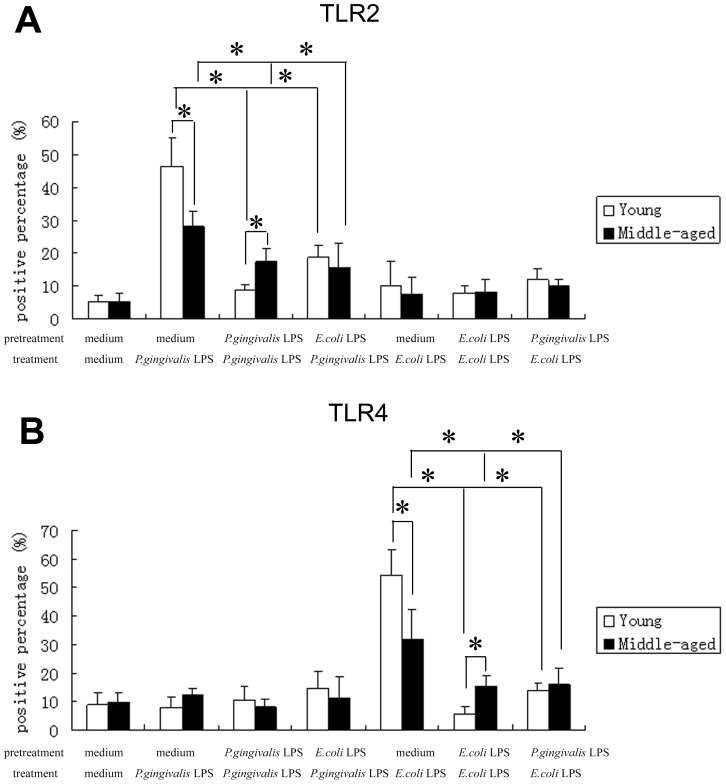
Protein expression changes of TLR2, 4 in peritoneal macrophages from young and middle-aged mice stimulated with LPS. Peritoneal macrophages from both young and middle-aged mice were stimulated with medium or LPS as described in the legends to Figure 1. Flow cytometry was used to quantify TLR2 (4A) and TLR4 (4B) protein expression levels. Data are expressed as mean±SD (n = 5 per group). *P<0.05.

**Figure 4:**
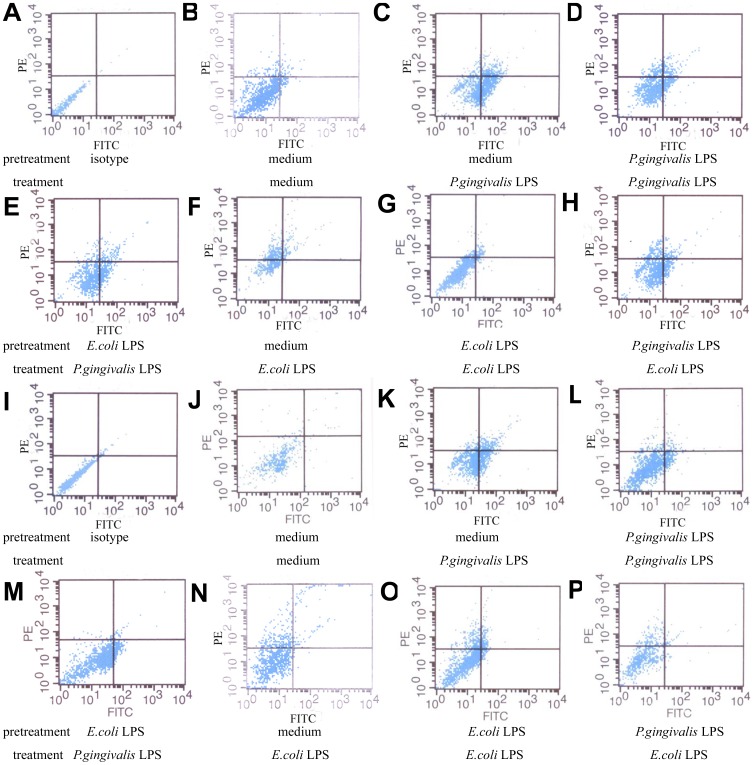
Protein expression of TLR2, 4 in peritoneal macrophages from young and middle-aged mice stimulated with LPS detected by flow cytometry. Peritoneal macrophages from both young (3A-3H) and middle-aged mice (3I-3P) were stimulated with medium or LPS as described in the legends to Figure 1. Protein expression levels of TLR2 (FITC-conjugated) and TLR4 (PE-conjugated) were detected by flow cytometry. One representative result of five independent experiments is shown.

We next examined whether the impaired ability of middle-aged mice to develop endotoxin tolerance was associated with the age-related changes in TLR2, 4. Quantitative real-time PCR analysis of total RNA and flow cytometry detection of TLR2, 4 surface expressions demonstrated that without stimulation, there were no significant differences in the mRNA and protein expressions of TLR2, 4 between peritoneal macrophages from young and middle-aged mice (p>0.05). *P. gingivalis* LPS stimulation led to the significant increases in the mRNA and protein expressions of TLR2 in macrophages from both young and middle-aged mice (p<0.05), and the expression levels in the cells from young mice were much higher than those from middle-aged mice (p<0.05). Similar to the expressions of TLR2, in macrophages from young and middle-age mice upon *E. coli* LPS stimulation, marked increases in the expressions of TLR4 mRNA and protein could also be observed (p<0.05), and the expression levels of TLR4 in the cells from young mice were significantly higher than those from middle-aged mice too (p<0.05) (Figure 2, 3). The representative result of five independent flow cytometry detections was shown in Figure 4.

Furthermore, the reduced mRNA and protein expressions of TLR2 or 4 were observed in macrophages from both young and middle-aged mice after *P. gingivalis* LPS or *E. coli* LPS restimulation respectively (homotolerance) (p<0.05), and the expression levels of TLR2, 4 in the cells from young mice were significantly lower than those from middle-aged mice (p<0.05) (Figure 2, 3, 4).

Our results also revealed the down-regulation of TLR2, 4 protein in macrophages from both young and middle-aged mice after different LPS restimulation (heterotolerance) (p<0.05). However, in heterotolerance groups, there were no significant differences in TLR2, 4 protein expressions between macrophages from young and middle-aged mice (p>0.05). In addition, in macrophages from young mice, but not middle-aged mice, the protein expressions of TLR2, 4 after repeated stimulation with the same LPS (homotolerance) were much lower than those after different LPS restimulation (heterotolerance) (p<0.05) (Figure 3, 4).

## Discussion

The primary etiologic factor of periodontitis is bacterial biofilm. Accumulating evidence indicates that specific microorganisms in subgingival plaque, including *P. gingivalis, P. intermedia,F. nucleatum* and *A. actinomycetemcomitans*, initiate the disease [26]. LPS is one of the most important virulence factors of gram-negative bacteria, and plays an essential role in triggering periodontal inflammation [27], [28]. Endotoxin tolerance induced by repeated LPS stimulations could lead to the reprogramming of the immune system, such as the downregulation of TNF-α and IL-1β, and the preservation of IL-10. It could play a protective role against inflammatory tissue destruction [29] and might have an effect on the development of periodontitis. Aging, which is characterized by the gradual decline in immune function, might also be associated with the prevalence and severity of periodontitis, at least in part [30], [31]. However, the influences of aging on endotoxin tolerance induced by periodontal pathogens and their underlying mechanisms still remain poorly characterized.

This is the first report on the effects of aging on endotoxin tolerance induced by LPS derived from periodontal bacteria. Our results provided evidence that the ability to develop tolerance in response to the repeated stimulation with LPS from both periodontal bacteria and non-periodontal bacteria was impaired in peritoneal macrophages from middle-aged mice. In addition, the different sensitivity to repeated LPS exposure in the cells from young and middle-aged mice might be partly associated with the different expressions levels of TLR2, 4.

In this present study, endotoxin tolerance was induced by *P. gingivalis* LPS and *E.coli* LPS. Although there are many common grounds on the biochemical and immunobiological properties of LPS from different gram-negative bacteria, differences in biological potency and pathogenicities still exist. It is not surprising that there are quantitative and/or qualitative differences in triggering TLRs and developing endotoxin tolerance between *E.coli* LPS and *P. gingivalis* LPS. *E.coli* LPS represents the classic LPS derived from gram-negative bacteria and is the optimal TLR4 agonist. LPS from many periodontopathic bacteria, such as *F. nucleatum* and *A. actinomycetemcomitans*, can also activate TLR4, and there are some similarities in triggering inflammation between *E. coli* LPS and these periodontopathic bacteria LPS [32], [33]. *P. gingivalis* LPS is an unusual pattern recognition receptor ligand for the innate defense system and expresses a low level of endotoxic activity relative to *E. coli* LPS. The protein structure of *P. gingivalis* LPS lacks heptose and 2-keto-3-deoxyoctonate, which are unique to enterobacterial LPS. Moreover, its lipid A exhibits a phosphorylation and acylation pattern, and contains branched and relatively longer fatty acids (15–17 carbon atoms). It is a TLR2 agonist, and its biochemical and immunobiological properties are quite different from *E. coli* LPS [34], [35]. Therefore, we chose two different LPS, *P. gingivalis* LPS and *E. coli* LPS, as the stimulators in this study to explore the reprogramming of the immune system resulted from endotoxin tolerance, which might take place in the development of periodontal inflammation.

Secretion of pro-inflammatory cytokines, including TNF-α, IL-1β and IL-6, and chemokine, such as IL-8, is one of the most important strategies utilized by the host to resist periodontal microorganisms. However, an orchestrated balance of pro-inflammatory cytokine, chemokine and anti-inflammatory cytokine release is critical for an innate immune response sufficient to resist periodontopathic bacteria without excessive damage to periodontal tissues. Endotoxin tolerance is an important protective mechanism, which could lead to the reprogramming of cytokine network to limit immune damage and maintain periodontal homeostasis. Therefore, it might be closely related to the progression of periodontitis. The impaired ability to develop endotoxin tolerance in old persons might be associated with the incontrollable periodontal inflammation.

Our findings demonstrated the diversity in different cytokine production after repeated LPS stimulations. Therefore, endotoxin tolerance is a case of reprogramming and immunomodulation rather than a global downregulation of cytokine expression and function. It is often linked with up-regulated expression of anti-inflammatory cytokines, such as IL-10 and TGF-ß, which contribute to the deactivation of monocytes/macrophages and the suppression of pro-inflammatory cytokine production in these cells [36], [37]. Our research indicated the higher expression levels of IL-10 in peritoneal macrophages from young mice after second LPS challenges compared with those in middle-aged mice, which might be responsible for the more excellent ability to develop endotoxin tolerance in the younger.

Periodontitis is an inflammatory disease resulted from polyinfection and there are many other virulence factors in periodonpathic bacteria than LPS. Therefore, heterotolerance might be more universal than homotolerance in the developmemt of periodontitis. Our results indicated that the heterotolerance was much weaker than homotolerance at the levels of TNF-α, which was consistent with previous research partially [7], but the interesting finding was that there were no significant differences between homotolerance and heterotolerance at the levels of IL-10, which disclosed the complexity of reprogramming in endotoxin tolerance.

Another finding of our study was that there were no effects of aging on the expression levels of TLR2, 4 in murine peritoneal macrophages without any stimulation. Our results are consistent with some previous studies. In Murciano’s researches, no significant differences between aged and young donors were observed on cell surface TLR2, 4 and 6 expression on lymphocytes, monocytes and granulocytes [38]. Similar finding was also reported in paper concerning TLR4 expression on macrophages from older and younger mice [39]. In addition, the decreased secretions of TNF-α and IL-10 by peritoneal macrophages from middle-aged mice stimulated with *E.coli* LPS or *P.gingivalis* LPS indicated the impaired functions of TLR2, 4 in these cells, which might be associated with the age-related changes in TLR2, 4 signaling transduction.

TLR2, 4 signaling pathway involves a cascade of intermediates, including myeloid differentiation factor-88 (MyD88), IL-1 receptor-associated kinase (IRAK) and TNF receptor-associated factor-6 (TRAF-6). Signaling transduction triggered by TLR2, 4 ligands leads to the activation of two distinct signaling pathways. One pathway leads to the activation of activator protein-1 through mitogen-activated protein kinase (MAPK), and the other enhances the activity of inhibitor of nuclear factor-_k_B kinase complex, which induces the release of nuclear factor-_k_B (NF-_ k_B) and the expression of cytokines and chemokines [40].

TLR2, 4 signaling transductions are so complicated that any signaling molecule in these pathways might have influences on the production of cytokines and chemokines. Therefore, it is hypothesized that there might be age-dependent changes in intermediates in TLR2, 4 signaling pathways, which might be associated with the differences in the function of TLR2, 4 between peritoneal macrophages from young and middle-aged mice. In an early research, Boehmer disclosed that levels of activated MAPKs did not differ by age in unstimulated macrophages, but LPS-stimulated macrophages from aged mice had <70% activated p38 and JNK than those of young controls, which might be related to the decreased production of proinflammatory cytokines, such as TNF-α and IL-6, in old mice [39]. Several years later, a gene expression microarray study from Chelvarajan indicated that aging influenced several downstream signaling molecules. It was found in his study that in macrophages from old mice, MyD88 and TRAF6 gene expressions were decreased and the levels of NF-κB components, p50, p52 and p65, were also reduced. However, the gene expression levels of negative regulatory molecules, Toll-interleukin-1 receptor–associated-protein (TIRAP) and IRAK-M, were enhanced. These findings contributed to disclosing the molecular basis of cytokine dysregulation in aged mice [41]. Therefore, age-associated alterations in signaling pathways might be responsible for the age-related deterioration of TLR2, 4. In contrast to these studies, there were some other researchers who believed that aging critically impaired intrinsic adaptive T-cell function, but preserved TLR-mediated immune responses [42].

Based on these understandings, we attempted to further explore the involvement of TLR2, 4 in endotoxin tolerance. Our study disclosed the less decreased expressions of TLR2, 4 and proinflammatory cytokine and the less enhanced production of anti-inflammatory cytokine in peritoneal macrophages from middle-aged mice treated with the second LPS stimulation, which implied that TLR2, 4 might interfere with the ability of the host to develop endotoxin tolerance. Our results were consistent with some early researches. Wang and Kim indicated that pretreatment of monocytes/macrophages with LPS or the other virulence factors strongly inhibited TLR2 or 4 activation in response to subsequent stimulation, then they drew the conclusion that tolerance could develop through down-regulation of TLR2 or 4 expression [9], [10].

Signaling through TLR2 and (or) 4 is one of the principal molecular mechanisms for the detection of gram-negative bacteria and their virulence factors by host cells. As discussed above, the effects of aging on TLR2, 4 signaling are so complicated that TLR2 and 4 are not the unique regulatory components that can modulate the development of endotoxin tolerance. Downstream signaling pathways and native cytokine profiles are also involved in the regulation of endotoxin tolerance. Recent in vitro studies have identified that impairment of IRAK activity and defects in the activation of MAPKs and NF-_k_B were associated with endotoxin tolerance in mouse macrophages and human monocytes [43]–[45]. New insights into the regulation of inflammation also disclosed the molecular mechanism of endotoxin tolerance, which involved some novel signaling molecules, such as protein kinase R (PKR) and phosphatidylinositol-3′-kinase (PI3K) [46]–[48]. In addition, our study revealed that even if the heterotolerance was much weaker than homotolerance at the levels of TNF-α, the surface expressions of TLR2, 4 in heterotolerance were not always much higher than those in homotolerance in both age groups. Therefore, we presumed that the age-dependent changes in some other signaling molecules in TLR2, 4 signaling pathways might be associated with the impaired ability of the host to develop endotoxin tolerance and heterotolerance might develop through the regulation of the downstream signaling pathways other than down-regulation of TLR2, 4 expression.

Periodontal infections are polymicrobial in nature, and numerous virulence factors are involved in it. The effects and mechanisms of heterotolerance in periodontal tissues might be more complex than those in vitro. Up to now, most of the studies about endotoxin tolerance are in vitro. Even though there were some in vivo researches concerning homotolerance [49], [50], reports on in vivo heterotolerance are very scarce and it is still not clear the effects of aging on endotoxin tolerance in vivo. Astiz’s early research demonstrated the reduced concentrations of IL-6, IL-8 and TNF-α in normal human volunteers pretreated with lipid A and treated with *E. coli* LPS [51]. Several years later, Lehner developed a mice model challenged with LPS and restimulated with serovar Typhimurium. In his research, an attenuation of cytokine production (TNF-α, IFN-γ and IL-6) and an increased capacity to recruit neutrophilic granulocytes in tolerant mice were revealed [52]. However, the mechanisms of heterotolerance in vivo still need to be explored.

In summary, the present study showed that peritoneal macrophages from middle-aged mice were more sensitive to repeated LPS stimulations, and the impaired ability to develop endotoxin tolerance might be related to the less decreased expressions of TLR2, 4. The effects of aging on tolerance in vivo might be much complicated. Therefore, further investigations of different sensitivity between young and aged subjects to repeated bacteria exposure are necessary for a better understanding of immune mechanisms of periodontitis in older adults.

## Materials and Methods

### Ethics Statement

This study was approved by the Ethical Committee of Nanjing Medical University (Permit Number: 2010-018) and all experiments were performed in agreement with national regulations on animal welfare and animal experiments. All efforts were made to minimize suffering.

### Animals

Young (2-month-old) and middle-aged (12-month-old) ICR mice were purchased from Vital River (Beijing, China) and maintained in an environmentally controlled facility at 21°C under a 12 h light : 12 h dark cycle.

### Reagents

*P. gingivalis* ATCC 33277 LPS and *E. coli* O127:B8 LPS were purchased from Invivogen (CA, USA) and Sigma Aldrich (Missouri, USA) separately. ELISA kit was obtained from Biosource (CO, USA). SYBR Premix Ex Taq was purchased from Takara (Dalian, China), and FITC-conjugated anti-TLR2 antibodies, PE-conjugated anti-TLR4 antibodies, FITC-conjugated IgG2b isotype control and PE-conjugated IgG1 isotype control were obtained from eBioscience (CA, USA).

### Cell Culture and LPS Stimulation

1.5 ml 4% thioglycollate broth (Sigma, USA) was injected intra-peritoneally in ICR mice. Three days later, the mice were sacrificed. Peritoneal macrophages were isolated by lavage of the peritoneal cavity with cold phosphate-buffered saline (PBS) and collected by centrifugation. Cells were then cultured in RPMI 1640 (GIBCO, USA) supplemented with 10% fetal calf serum (Hyclone, USA) at a concentration of 2×10^6^ cells/ml in 6-well plates. 2 h later, non-adherent cells were discarded. The remaining adherent cells were maintained in culture for 24 h until they were used for experiments [53].

Peritoneal macrophages from both young and middle-aged mice were divided into seven groups separately (n = 5 per group). Group 1 was cultured in medium alone. Groups 2 and 5 were treated with medium for 24 h, washed three times with fresh medium and stimulated with 1 µg/ml *P. gingivalis* LPS or 1 µg/ml *E. coli* LPS respectively for 6 h to determine TLR2, 4 mRNA or 24 h for analysis of TLR2, 4 proteins and cytokines. Group 3, 4, 6 and 7 were incubated for 24 h with an initial LPS challenge (1 µg/ml *P. gingivalis* LPS?or 1 µg/ml *E. coli* LPS), washed, and then restimulated with either 1 µg/ml *P. gingivalis* LPS or 1 µg/ml *E. coli* LPS for the subsequent detections as described in group 2 and 5. All cells were washed three times with cold PBS and collected for real-time PCR and flow cytometry analysis. Cell-free supernatants were harvested and stored at −20°C for cytokine assays.

### Cytokine Detection

Cytokine levels (TNF-α and IL-10) in the culture supernatants were measured by ELISA according to the manufacturer’s instruction. The plates were read in an ELISA-reader (Bio-Hit, Helsinki, Finland) at 490 nm.

### Real-time PCR

Total RNA was prepared from peritoneal macrophages, which were stimulated with LPS or medium. cDNA was synthesized using a reverse transcription kit (Takara, China). Levels of ß-actin mRNA served as internal controls. The primer sequences were as follows (F/R): TLR2 (GAGTCAGACGTAGTGAGCA/AGTGTTCCTGCTGATGTCAAG); TLR4 (GCAGCAGGTGGAATTGTATC/TGTTCTCCTCTGCTGTTTG); and ß-actin (GCTACAGCTTCACCACCACAG/GGTCTTTACGGATGTCAACGTC).

Real-time PCR analysis was performed in duplicates in an ABI PRISM 7300 Real-Time PCR System (Applied Biosystems, USA). The reaction product was quantified by the standard curve method [54]. A standard curve with predetermined concentrations and serial diluted respective PCR amplification products from 1×10^−1^ to 1×10^−10^ was constructed for each transcript analyzed.

### Flow Cytometry

Peritoneal macrophages were scraped off from 6-well plates and washed in washing buffer (PBS containing 1% fetal calf serum). To analyze TLR2, 4 surface expressions, cells were incubated with FITC-conjugated anti-TLR2 antibodies and PE-conjugated anti-TLR4 antibodies for 30 min at 4°C in the dark. Corresponding isotypes to the above antibodies, conjugated to the appropriate fluorochromes, were used as controls for nonspecific binding of antibodies. After this incubation, cells were washed twice in washing buffer and then fixed in 1% formalin in PBS [55]. Expressions of TLR2, 4 on 10,000 viable cells were then gated and analyzed by a FACSCalibur (BD Biosciences,USA).

### Statistical Analysis

Statistical analysis of ELISA data was performed using ANOVA and Dunnett’s T3 test was used to compare differences between groups. The data of real-time PCR and flow cytometry were analyzed using the Kruskal–Wallis test, and subsequently the Mann–Whitney test was performed as a post-hoc test. All data are presented as means±SD. The level of significance was set at p<0.05.
